# Pulmonary Cryptococcosis: A Diagnostic and Management Challenge Case Report

**DOI:** 10.7759/cureus.59361

**Published:** 2024-04-30

**Authors:** Kylee Cardoso, Lisa Carroll

**Affiliations:** 1 Infectious Disease, Edward Via College of Osteopathic Medicine-Carolinas, Spartanburg, USA; 2 Family Medicine, Edward Via College of Osteopathic Medicine-Carolinas, Spartanburg, USA

**Keywords:** cryptococcus gattii, fungal infection, pneumonia, cryptococcosis, pulmonary cryptococcus, cryptococcal pneumonia, india ink, pulmonary cryptococcosis, cryptococcus neoformans (c. neoformans), pulmonary cryptococcal infection

## Abstract

Cryptococcosis is a fungal infection that may arise in immunocompromised or immunocompetent individuals. This case report seeks to demonstrate the difficulty in diagnosing and treating cryptococcosis based on clinical presentation and radiographic features as together, they mimic other pathological conditions. A 56-year-old female with cirrhosis presented with persistent abdominal pain, dyspnea, vomiting, and diarrhea and was diagnosed with pulmonary cryptococcosis after an initial diagnosis of bacterial pneumonia. With no improvement following antibiotic therapy for suspected bacterial pneumonia, additional imaging was performed with a confirmatory lung biopsy for pulmonary cryptococcosis. The patient initiated antifungal therapy with the anticipation of completing approximately 12 months with follow-up imaging to evaluate improvement. After the patient experienced adverse effects of antifungal therapy and did not achieve significant improvement or recovery in her condition, it was apparent that cryptococcal pneumonia presents both diagnostic and management challenges that must be further explored.

## Introduction

*Cryptococcus* is a fungus in the soil, bird droppings (especially pigeons), and decaying wood most often located geographically in the Northwestern United States and Vancouver Island in Canada [1]. The two species associated with infection via the inhalation of spores through the respiratory tract in humans are *Cryptococcus neoformans* and *Cryptococcus gattii* [2]. Humans are commonly exposed to *Cryptococcus neoformans* by the time they attend school. Therefore, their immune systems have usually mounted antibodies against the pathogen by that time [2]. While the yearly incidence of cryptococcosis in the United States is between 0.4 and 1.3 cases per 100,000 people, the risk of developing cryptococcosis is greater in those with risk factors indicative of compromised immune function [3]. These risk factors include, but are not limited to, human immunodeficiency virus (HIV)-positive individuals, those with acquired immunodeficiency syndrome (AIDS), those with conditions being managed with immunosuppressive therapies, and those with cirrhosis [4]. It is important to note that cryptococcal infection also occurs in immunocompetent individuals, albeit immunocompromised hosts have a higher susceptibility.

Symptomatic cryptococcosis typically manifests as a pulmonary infection and/or disseminated meningitis. The most common presenting symptoms include fever, headache, cough, dyspnea, and generalized fatigue. Additional features that are more suggestive of disseminated cryptococcosis include neck stiffness, photophobia, and altered mental status [5].

The imaging features of cryptococcal infection tend to mimic other pathological conditions. A case report reviewing the presentation among two immunosuppressed patients and one immunocompetent patient revealed the difficulty in diagnosing cryptococcal pneumonia based on radiological findings alone [6]. One immunocompromised patient presented with nodular opacities in bilateral lung fields on chest X-ray and enlarged lymph nodes in the mediastinum and axillary regions on CT scan [6]. A CT scan of the thorax in the second immunocompromised patient demonstrated a mass in the right lower lobe of the lungs mimicking a malignant lesion [6]. In contrast, the immunocompetent patient presented with ring-enhancing lesions with surrounding edema on a CT scan of the brain and enlarged mediastinal lymph nodes on a CT of the thorax [6]. Consequently, due to its rare incidence, nonspecific clinical presentation, and radiological features, cryptococcosis is most often initially misdiagnosed as a lung malignancy, tuberculosis, or bacterial pneumonia [3].

The definitive diagnosis of cryptococcal meningitis is made by performing a lumbar puncture and evaluating the cerebral spinal fluid [5]. India ink, a special stain that allows the visualization of encapsulated yeasts, indicates the presence of *Cryptococcus* in the spinal fluid and should be utilized along with fungal culture, cryptococcal antigen testing, and the measurement of opening pressure. The predictors of cryptococcal meningitis on spinal fluid evaluation include an opening pressure of greater than 25 cm of water, low glucose, high protein, normal or elevated white blood cell count with lymphocyte predominance, positive India ink stain, and positive antigen testing [5]. Pulmonary cryptococcosis is diagnosed based on radiological and histopathological findings performed with procedures such as lymph node sampling and image-guided biopsies [6]. Encapsulated yeasts visualized within lung tissue histologically confirm pulmonary cryptococcosis along with positive cryptococcal antigen testing [6]. It is crucial to rule out cryptococcal meningitis by performing a lumbar puncture in patients experiencing neurologic deficits and/or in immunocompromised patients [7].

The mainstay management of cryptococcosis consists of antifungal therapy. Azole antifungals, including fluconazole, interfere with fungal metabolism, thereby inhibiting the formation of cell membranes [8]. The adverse effects of antifungal therapy may include corrected QT (QTc) prolongation, maculopapular skin rashes, and hepatotoxicity [8]. Individuals with cryptococcal meningitis have a high mortality risk if not managed with therapy rapidly, as infection in the central nervous system causes the inflammation of brain tissue and increased intracranial pressure [2]. HIV-infected patients may additionally follow a more rigid regimen including maintenance therapy.

## Case presentation

**Figure 1:**
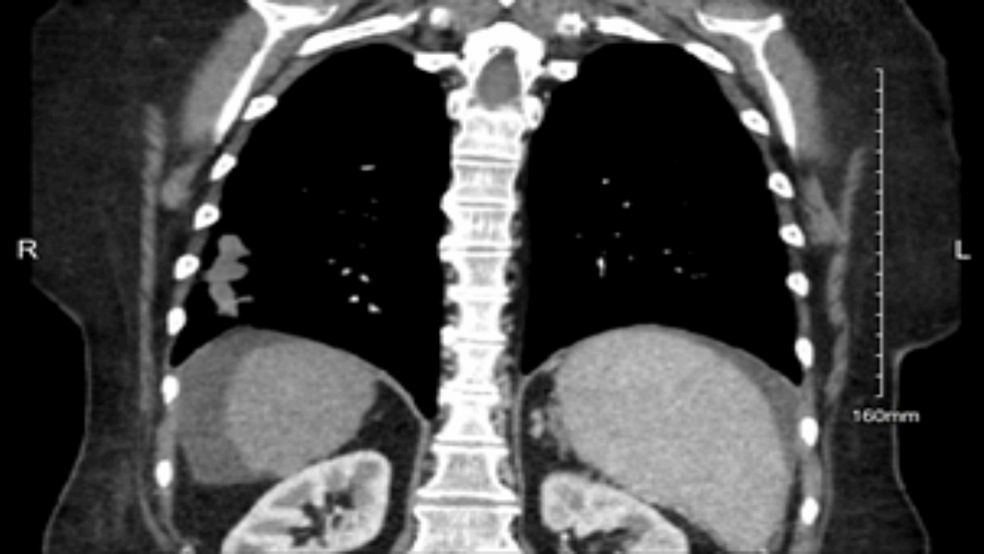
CT scan of the abdomen and pelvis with IV and oral contrast. Multiple pulmonary nodules and masses in the right middle and lower lobe of the lungs.

**Figure 2:**
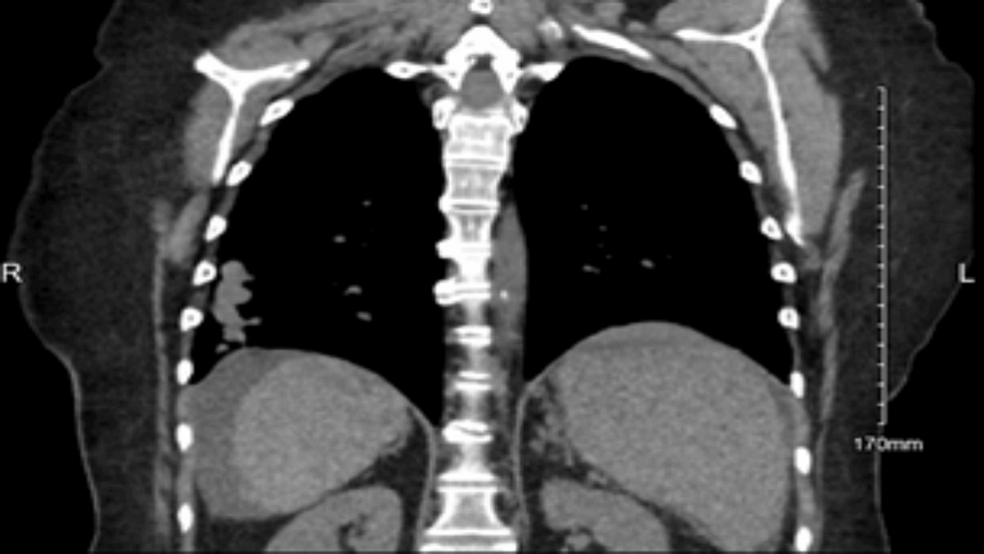
CT scan of the chest without contrast. No significant changes in the size or number of scattered pulmonary nodules from Figure 1.

A 56-year-old Caucasian female, with cirrhosis secondary to metabolic dysfunction-associated steatohepatitis, symptomatic cholelithiasis, and hypertension, presented to the emergency department with a primary concern of persistent abdominal pain. The patient stated that abdominal discomfort was a common symptom of her cirrhosis, but the pain had become acutely worse and constant within the past 48 hours. Associated symptoms included fatigue, dyspnea on exertion, poor appetite, vomiting, and diarrhea. The patient did not have a recent travel history but admitted to growing up with birds throughout her life. Her physical examination only revealed that the patient appeared uncomfortable but nontoxic and not in acute distress. Abdominal examination revealed a soft, non-tender abdomen with normal bowel sounds. There was no rebound tenderness, guarding, organomegaly, or masses on palpation. The patient was discharged with a referral to outpatient gastroenterology, as well as an interventional radiology consultation two weeks later due to prior imaging findings. Two months prior to her emergency department visit, a CT scan of the abdomen and pelvis with IV and oral contrast was ordered by her primary care provider after concern for possible pancreatitis. The imaging ordered by her primary care provider showed multiple pulmonary nodules and masses in the right middle and lower lobe of the lungs as seen in Figure 1, suggestive of an infectious or inflammatory pneumonia, which her pulmonologist managed with antibiotic therapy. A repeat CT scan of the chest without contrast one month after antibiotic therapy noted no significant changes in the size or number of the scattered pulmonary nodules as seen in Figure 2. Interventional radiology performed a CT-guided right lung needle biopsy. The histopathology of the biopsy revealed necrotic debris with encapsulated fungal yeasts morphologically suggestive of *Cryptococcus*. The patient was admitted to the hospital to rule out cryptococcal meningitis. A lumbar puncture was performed and within normal limits with an opening pressure of 15 cm of water, no white blood cells, and normal protein. India ink staining was negative, and fungal cultures were not obtained. HIV testing and cryptococcal antigens were negative. With the results of the lumbar puncture, cryptococcal meningitis was not suspected, and the patient was ultimately diagnosed with serum antigen-negative but biopsy-positive pulmonary cryptococcosis.

**Figure 3:**
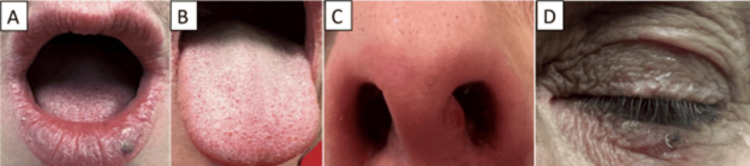
Adverse effects of fluconazole therapy. Mucocutaneous lesions on the (A) lower lip, (B) tongue, (C) nasal vestibule, and (D) periorbital skin.

Per infectious disease, the patient was started on 400 mg daily of fluconazole to manage her pulmonary cryptococcosis with the anticipation of approximately a 12-month course and follow-up imaging to assess improvement. Upon the initiation of fluconazole therapy, the patient began experiencing adverse effects. The patient developed mucocutaneous lesions on her lips, tongue, periorbital skin, and nasal vestibule as illustrated in Figure 3. The lesions were managed with a topical corticosteroid, which alleviated some of the discomfort. However, the lesions persisted for the entire duration of fluconazole therapy.

**Figure 4:**
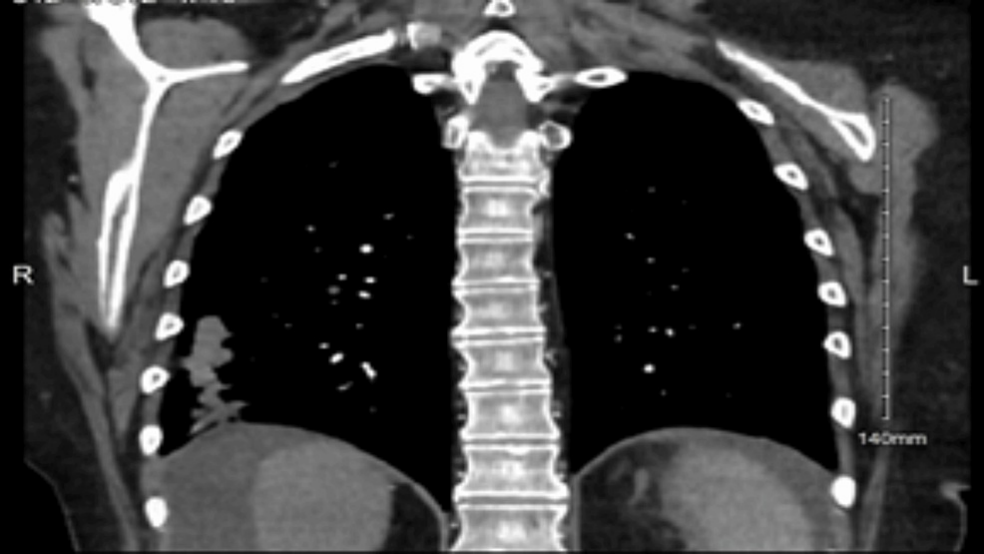
CT scan of the chest with contrast. Persistent scattered and clustered nodules. No significant change in size or number from Figure 1 and Figure 2.

At nine months into management with fluconazole therapy, a CT scan of the chest with contrast revealed no significant changes the in size or number of the pulmonary nodules as seen in Figure 4, in comparison to the patient’s prior CT scans. Per infectious disease, continuing fluconazole therapy would not be clinically beneficial for the patient. Fluconazole therapy was discontinued, the patient’s symptoms were to be monitored, and repeat imaging would be obtained in three months.

## Discussion

The management of pulmonary cryptococcosis remains variable as an optimal treatment method continues to be under investigation. Oral fluconazole 400 mg (6 mg/kg) taken daily for six to 12 months is the typical regimen in patients diagnosed with non-severe pulmonary cryptococcosis [9]. Patients positive for HIV most often continue with maintenance therapy consisting of 200 mg daily of fluconazole for approximately one year after treatment as long as their viral loads are suppressed, cluster of differentiation 4 (CD4) count is >100, and cryptococcal antigen titers remain stable [10]. Without management with antifungal therapies, cryptococcosis may progress leading to life-threatening complications. Seizures, altered states of consciousness, coma, and death are a few consequences of cryptococcosis progression without management in immunocompromised patients [5].

Further research is necessary in order to address optimal management leading to resolution on follow-up radiographic imaging. Whether antifungal therapy provides the most benefit in managing pulmonary cryptococcosis remains controversial, especially when managing infected asymptomatic immunocompetent patients. In a retrospective review of 76 patients with confirmed pulmonary cryptococcosis, 71 cases showed total recovery, four cases showed improvement, and one case died secondary to progression into cryptococcal meningitis [11]. Fluconazole monotherapy was the management of choice for 39 patients, while 25 immunocompetent asymptomatic patients did not receive any therapies and were merely observed [11]. Twenty-four of the 25 patients observed reached complete recovery, while one patient showed improvement radiographically [11]. While all the patients managed with fluconazole monotherapy reached complete recovery, the results of this study suggested that observation alone may be a reasonable management option in some patients [11]. Ultimately, clear guidelines would need to be established to determine the appropriate population of patients that would benefit from observation only. This would require more research focused on assessing management outcomes, as well as risk versus benefit and ethical considerations of not managing all patients with antifungal therapy.

The case presented in this report demonstrated an initially missed diagnosis of pulmonary cryptococcosis, which is seemingly common due to mimicking other pathologies clinically and radiographically. However, this case also had unique aspects in the presentation of pulmonary cryptococcosis. Geographically, this patient presented in the Southeastern United States without a history of recent travel, whereas cases of cryptococcosis are classically found in the Northwestern United States or following recent travel from an endemic area. Additionally, patients with acquired immunodeficiency syndrome are the main hosts susceptible to *Cryptococcus* spp., as it is an opportunistic pathogen [3]. Per 100,000 patients with AIDS, two to seven cases of cryptococcosis are diagnosed yearly in the United States [3]. This patient did not have AIDS but did have cirrhosis. According to one report, patients with cirrhosis who develop cryptococcosis have an 81% estimated mortality [12]. This may be largely due to delayed diagnosis and as a result the delayed onset of management [12]. Similarly, the patient presented in this case had a delay in diagnosis and initiation of therapy as her symptoms appeared initially to be exacerbations of cirrhosis and imaging suggested bacterial pneumonia. While the patient presented in this case continues to be managed, after 12 months of antifungal therapy, the pulmonary nodules remained stable. There was no reduction in size or number of nodules, suggesting neither improvement nor recovery of her condition. The patient’s experience of added side effects of therapy without the improvement of her condition raises the question of what the optimal management for pulmonary cryptococcosis in immunocompromised patients should be. With the mainstay of treatment not showing success in this case and making the patient symptomatically worse, further studies are needed to inform decisions regarding which patients should be managed with antifungal therapy and which patients should not.

In summary, the optimal management of pulmonary cryptococcosis in different immunologic populations remains controversial and requires further investigation. Determining which patients would benefit most from antifungal therapy versus which patients would benefit most from observation alone remains questionable.

## Conclusions

It is crucial that clinicians consider cryptococcal organisms in patients with nonspecific clinical presentations, especially in combination with radiographic features that may be suggestive of other etiologies. The case presented in this report demonstrated pulmonary cryptococcosis initially mimicking bacterial pneumonia radiographically. Moreover, once diagnosed, it is important to consider the likelihood of improvement and recovery of pulmonary cryptococcosis with antifungal therapy, the current mainstay management. Although mainstay, antifungal therapy can subject patients to adverse effects and does not always improve or promote recovery in all patients, as seen in this case. Ultimately, further research is necessary to focus on the best management plan for patients with pulmonary cryptococcosis, considering comorbidities, the burden of adverse effects, and the likelihood of improvement and recovery of the condition.
